# The repeatability of mating failure in a polyandrous bug

**DOI:** 10.1111/jeb.12678

**Published:** 2015-07-06

**Authors:** E. V(Ginny) Greenway, D. M. Shuker

**Affiliations:** ^1^ School of BiologyUniversity of St AndrewsSt AndrewsFifeKY16 9THUK

**Keywords:** insect reproduction, mating failure, polyandry, sexual selection

## Abstract

Mating failure, characterized by the lack of production of offspring following copulation, is relatively common across taxa yet is little understood. It is unclear whether mating failures are stochastic occurrences between incompatible mating partners or represent a persistent, meaningful phenotype on the part of one or other sex. Here we test this in the seed bug *Lygaeus simulans*, by sequentially mating families of males with randomly allocated unrelated females and calculating the repeatability of mating outcome for each individual male and family. Mating outcome is significantly repeatable within individual males but not across full‐sib brothers. We conclude that mating failure represents a consistent male‐associated phenotype with low heritability in this species, affected by as yet undetermined environmental influences on males.

## Introduction

Why do so many matings fail? Given that successful fertilization is the *raison d’être* of mating and should be a focus of both natural and sexual selection, relatively frequent failure to convert matings into fertilizations in the absence of competitors is something of an evolutionary enigma. The costs of both achieving and then carrying out matings to both males and females are well documented (Thornhill & Alcock, 1983; Wedell *et al*., 2002), and selection to capitalize on matings is apparent in the evolution of a huge diversity of genital and sperm morphologies and peri‐ and post‐copulatory behaviours (Eberhard, 1996; Simmons, 2001). Failure to find a suitable mate, due to demographic effects, low mate encounter rate, out‐competition by rivals or prereproductive death, has been well documented (Rhainds, 2010). However, matings that fail to result in offspring production despite the successfully securing of, and coupling with, a mate have achieved somewhat less attention until recently (e.g. Tyler & Tregenza, 2013). Studies and meta‐analyses across taxa reveal nontrivial levels of copulation failure, occurring on average in 22% of matings across 30 insect species (García‐González, 2004), and hatching failure rates of up to 55% in avian species (Koenig, 1982). However, the precise mechanisms for such failures and the fitness consequences for individuals remain unclear.

A mating can fail to produce offspring for myriad reasons and at any stage of copulation, and putative explanations of mating failure range from absence or depletion of sperm, mechanical intromission or insemination failures, genetic incompatibility, and the exertion of cryptic choice by females (Eberhard, 1996; García‐González, 2004). Given the degree of heritable genetic variation demonstrated in fertility‐related traits such as sperm morphology (Morrow & Gage, 2001; Evans, 2011; Murray *et al*., 2011), mating duration (MacBean & Parsons, 1967) and sperm competition success (Konior *et al*., 2006), natural selection should prevent the maintenance of genetically determined and costly low fertility within populations. Although it may theoretically be preserved at low levels by mutation‐selection balance (Lande, 1995), in actuality less fertile males would be outcompeted by more fertile rivals and so fail to gain paternity. Thus, such a deleterious trait should not persist in the population, although more ephemeral or environmentally determined forms of infertility such as sperm depletion or sperm limitation are unlikely to be so highly penalized by sexual selection and therefore might be more common (Sheldon, 1994; Hasson & Stone, 2009).

Measuring the repeatability of mating failure offers a method to tease apart some of these possibilities. For instance, by establishing how consistent male mating outcomes are over successive trials would help us to estimate the importance of female influence. Furthermore, repeatability can indicate the upper limit of heritability (Boake, 1989), providing an insight into the genetic components of behaviour upon which natural and sexual selection may act. Although high levels of repeatability of reproductive behaviour have been demonstrated in some species (e.g. sexual conflict outcome in *Coelopa frigida* (Shuker & Day, 2001)), other studies report little or none (e.g. Tregenza *et al*., 2009). Similarly, using related individuals to gauge trait heritability has produced opposing results in closely related species (Taylor *et al*., 2013; Bretman *et al*., 2014). Moreover, in their meta‐analysis Bell *et al*. (2009) observed that although components of mating success are generally repeatable, mate preference is far less so, reflecting higher levels of behavioural context dependence and condition dependence (e.g. Cotton *et al*., 2006). This broad range of repeatability and heritability, both within and between species and behaviours, has long been well appreciated and is likely in part to reflect varying genotype‐by‐environment interactions (GEIs). Using repeated measures from both individual male and family‐level male mating success, we aim to gain an indication of the genetic and environmental components of mating failure.

The study species, *Lygaeus simulans* (Heteroptera: Lygaeidae), is characterized by a promiscuous mating system in which both males and females engage in multiple and prolonged copulations. Rates of mating failure are nontrivial, ranging from 36% to 60% (Tadler, 1999a,b). Some virgin females (of both *L. simulans* and sister species *Lygaeus equestris*) produce infertile eggs, but far higher levels of oviposition are stimulated by mating (Sillén‐Tullberg, 1981). As precopulatory sexual selection appears to be relatively weak in the sister species *L. equestris* (Burdfield‐Steel *et al*., 2013; Dougherty & Shuker, 2014), understanding mating failures may be key in unravelling the extent of post‐copulatory sexual selection in this species. We used an experimental paradigm in which males were given successive matings with randomly selected virgin females. We firstly assessed to what extent mating outcome was repeatable within individual males, and secondly across full‐sib families. For mating failure to be considered a meaningful phenotype (i.e. not just a stochastic failure unrelated to male and/or female reproductive anatomy, physiology or behaviour), we would predict that it should demonstrate significant repeatability. Whether we would expect this repeatability to be maintained at the family level depends on the mechanisms underlying mating failure. Repeatability of outcome between full sibs would suggest a high level of genetic control, whereas little or no repeatability points towards a lack of heritability and substantial environmental influence.

## Materials and Methods

### Husbandry and production of full‐sib males

*Lygaeus simulans* fifth instar nymphs were collected from a laboratory stock population (originally collected from Tuscany in 2004) and raised to adulthood at a temperature of 29 °C and under a 22:2‐h light:dark cycle to prevent the initiation of reproductive diapause. Newly moulted adult virgin males and females were sorted by sex into plastic tubs with *ad libitum* organic sunflower seeds and tubes of distilled water with a cotton bung until sexual maturity. Sexually mature males and females were then randomly paired in individual tubs (with *ad libitum* seeds and water) and left to mate. The resulting eggs from these pairings were incubated and offspring‐collected. At least 10 full‐sib virgin males were collected from each family (*N *=* *14) and reared to sexual maturity at maximum densities of 10 individuals per tub.

### Mating trials

Individual focal males (*N *=* *168) between 7 and 10 days old were placed in 85‐mm‐diameter petri dishes and randomly paired with virgin females. Mating attempts were scored within the first two hours of continuous observation. After this period, dishes were checked at 10‐min intervals for new pairings (males and females in end‐to‐end mating position) or separations for a further 7½ h. Only matings over a cut‐off of 30 min were considered, as this constitutes the estimated minimum time required for sperm transfer to occur in this species (Micholitsch *et al*., 2000). Individuals were separated immediately after mating, pairings that had not terminated naturally by the end of the 9½‐h observation period were separated, and mating latency and duration were recorded.

This procedure was repeated for a further three successive days, with males randomly paired with a new virgin female each day. Males were returned to the incubator overnight between trials in petri dishes containing three sunflower seeds and water source. Only males that mated 3 or 4 times were included in analysis to calculate repeatability robustly (*N *=* *102).

After each mating trial, mated females were removed and placed in individual tubs with *ad libitum* sunflower seeds and distilled water and allowed to oviposit. If females produced eggs, these were incubated for a further 7 days, and if hatching and nymph production resulted, the mating was classified as successful. Failed matings were indicated by the absence of either eggs or offspring after this period.

### Statistical analysis

We classed mating outcome as a binary response (‘success’ or ‘failure’). Repeatability of mating outcomes was calculated using a binomially distributed GLMM with logit‐link and multiplicative overdispersion, with ‘male ID’ as repeatability ‘group’ (r package rptR: Nakagawa & Schielzeth, 2010). Repeatability of outcome within families was calculated similarly, replacing individual mating outcomes as the unit of repeatability with the sum of each individual's successful or failed matings and ‘family ID’ as the repeatability group. Here we report repeatabilities on the link scale (see Supporting Information for original scale repeatabilities). Similarly, due to their bimodal distribution (see Supporting Information) mating durations were classified by their position relative to the mean mating duration (295.05 min). As such, ‘short’ durations consisted of matings up to and including 295 min in length. Any matings exceeding 295 min were considered to be ‘long’ in duration.

## Results

**Figure 1:**
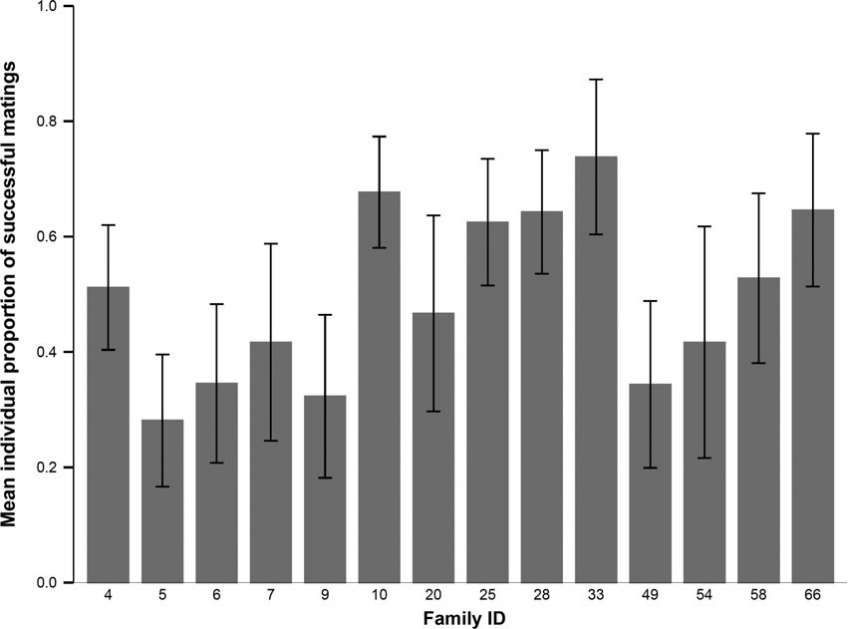
Mean proportion of individual mating success across full‐sib families. Bars denote ± 1 SE.

The mating failure rate was 49.2%; 23.6% of matings resulted in no eggs and 25.5% in no fertile eggs, and the majority of males mated on at least three of the available four opportunities (mean mating number = 3.04 ±  SE 0.102). Total mating success displayed a clearly bimodal distribution, with zero or 100% mating success representing two of the most frequent outcomes (see Supporting Information), and individual mating outcome was significantly repeatable (*R *=* *0.415 ± SE 0.07, *P *=* *0.001). On the other hand, whilst mean mating success varied considerably between the 14 families of males (Fig. 1), the repeatability of mating outcome within family was low and nonsignificant (*R *=* *0.007 ± SE 0.02, *P *=* *0.268). When any males that mated with a female who did not lay eggs were excluded from analysis, levels of repeatability increased individual male mating outcome repeatability (*n *=* *45, *R *=* *0.464 ±  SE 0.11, *P *=* *0.001). Longer copulations were significantly more likely to lead to successful matings (*χ*²_(1)_ = 63.42, *P* = <0.001), and mating duration, like mean mating success, exhibited high between‐family variation (see Supporting Information). Mating duration displayed low but significant levels of repeatability at the individual level (*R *=* *0.266 ± SE 0.07, *P *=* *0.001) and but no significant family repeatability (*R *=* *0.019 ± SE 0.02, *P *=* *0.124).

The number of previous matings males had undertaken had no significant effect on mating failure (GLMM, *Z *=* *1.056, *P *=* *0.291; see Supporting Information). Therefore, mating order had no effect on outcome, as a result of either possible sperm depletion or sexual experience.

## Discussion

Mating failure is repeatable for male *Lygaeus simulans* bugs. Despite acknowledgement that widespread permanent sterility or low fertility within populations has traditionally been considered unlikely (Sheldon, 1994; García‐González, 2004), approximately half of the matings in this study failed to result in fertilization and, more importantly, around a quarter of multiply‐mating males fathered no offspring at all. Although demonstration of high levels of mating failure within the Lygaeidae is not novel (Tadler, 1999b), this is the first time such failure has been shown to be significantly repeatable within individuals in any species.

The observed repeatabilities, comparatively high in the context of behavioural studies (Bell *et al*., 2009), suggest that mating failure constitutes a genuine male‐associated phenotype. By comparison, the low repeatabilities of copulation duration suggest perhaps that females play more of a role in determining copulation length. Explanations of mating failure in *L. simulans* have focused on intromittent organ insertion as well as potential female prevention of insemination (Tadler, 1999a). Tadler (1999b) provides evidence of stabilizing selection on male intromittent organ length, with males possessing an intromittent organ of intermediate length achieving highest levels of fertilization (see also Dougherty *et al*., 2015). Incidentally, intromittent organ length in the sister species *Lygaeus equestris* has been demonstrated to be significantly heritable (H^2^ = 0.306 ± SE 0.18; Higgins *et al*., 2009)**.** Additionally, mating duration correlates significantly with mating outcome both in this and previous studies (Micholitsch *et al*., 2000), with failed matings terminating typically within the initial hour of mating. Despite this, duration displays a lower level of repeatability within individuals than mating outcome, suggesting it is not the sole determinant of mating failure. The absence of a significant effect of mating order also indicates no significant role of sperm limitation in mating failure in this species.

Examining mating outcome repeatability at the family level offers further mechanistic insight. Although repeatability provides an upper limit to heritability, it does not confirm that level of heritability (Boake, 1989). The lack of congruence between our individual and family‐level repeatabilities emphasizes this, with the discrepancy suggesting that nonadditive genetic or environmental effects influence variation in mating failure. As selection acts solely upon heritable variation, this goes some way to explaining why mating failures persist despite selection acting to maximize reproductive success, although it should be noted that the relatively small family sample size used limits our ability to reject definitively the possibility of a genetic component to mating failure. In terms of environmental effects, arguably the most important factor likely to influence a male's mating success is that of the female with whom he is mating (Eberhard, 1996; Ingleby *et al*., 2010). However, the repeatability of mating outcome displayed by individual males mating with multiple independent females precludes variation among females in terms of their ‘copulatory’ environment influencing our results and leads us to conclude that environmental effects associated more directly with males are the predominant source of phenotypic variation. What these are, and to what extent they are associated with laboratory rearing for multiple generations, remains to be seen.

The vast majority of mating behaviour studies measure individuals only once, and the degree to which behavioural measurements are repeatable within individuals remains to be verified in most species. Estimating the repeatability of mating failure is important not only for our understanding of this enigmatic trait but also in terms of experimental design and interpretation. Overlooking mating failure can result in incorrect interpretations of sperm competition experiments and incorrect estimates of polyandry (García‐González, 2004), both of which are central to post‐copulatory sexual selection research. Put another way, we should not necessarily discard individuals or pairs from our analyses when mating has apparently ‘failed’. More positively, significant consistency of mating outcome and no apparent effect of virgin male status provide support for the results of previous studies on this species performed using single mating trials.

Although it is possible that laboratory conditions may have generally lessened the selection pressure on male mating performance in *L. simulans*, the level of repeatability demonstrated in this study confirms that mating failures cannot be dismissed as stochastic events or purely the result of male–female preference or genetic incompatibilities. Such high levels of male infertility and variation in reproductive success would intuitively have substantial repercussions for mating strategy evolution, increasing the likelihood of female sperm limitation (Wedell *et al*., 2002) and as a result female remating rates (Sheldon, 1994; Jennions & Petrie, 2000; Hasson & Stone, 2009). The risk of mating failure, even if low, has been both theoretically and empirically demonstrated to support fertility insurance and bet‐hedging strategies (Forbes, 2014; García‐González *et al*., 2014). Under these conditions, direct benefits derived by polyandrous individuals, in terms of maximizing offspring production, are likely to outweigh the potential costs of mating multiply. Although mating failure is probably a complex trait, varying in origin across taxa, its prevalence suggests it should not be overlooked as a major factor in unravelling the evolution of polyandry.

## Supporting information


**Table S1** Link and original scale repeatabilities
**Table S2** Effect of mating order on outcome
**Figure S1** Frequency distribution of total individual mating success.
**Figure S2** No effect of mating order on mating failure.
**Figure S3** Frequency distribution of mating durations.
**Figure S4** Mean mating duration by family.
