# Supplementary material for: The repeatability of mating failure in a polyandrous bug
Source: J Evol Biol. 2015 Jul 6;28(8):1578–82. doi: 10.1111/jeb.12678 (PMC4744990; doi:10.1111/jeb.12678)
Supplement: Supplementary file 1 — Table S1 Link and original scale repeatabilities Table S2 Effect of mating order on outcome Figure S1 Frequency distribution of total individual mating success. Figure S2 No effect of mating order on mating failure. Figure S3 Frequency distribution of mating durations. Figure S4 Mean mating duration by family. [file JEB-28-1578-s001.pdf]

Supporting information

Table S1 Link and original scale repeatabilities- produced using rptR statistics package. See Nakagawa & Schielzeth (2010) for further description of the rptR package and link-scale and original scale repeatabilities

Repeatability	Level	Link-scale R ± S.E.	P-value	Original scale R ± S.E.	P-value
**Mating outcome**	Individual	0.415 ± 0.069	0.001	0.369 ± 0.065	0.001
	Family	0.007 ± 0.017	0.268	0.006 ± 0.014	0.268
**Mating duration**	Individual	0.266 ± 0.066	0.001	0.229 ± 0.058	0.001
	Family	0.019 ± 0.023	0.124	0.016 ± 0.019	0.124

Table S2 Effect of mating order on outcome - summary of GLMM output with binomial distribution, fitting mating number as a fixed effect and individual ID as a random effect

	Estimate	Std. error	Z value	Pr(>|z|)
**(Intercept)**	-0.2910	0.3515	-0.828	0.408
**Mating number**	0.1294	0.1226	1.056	0.291

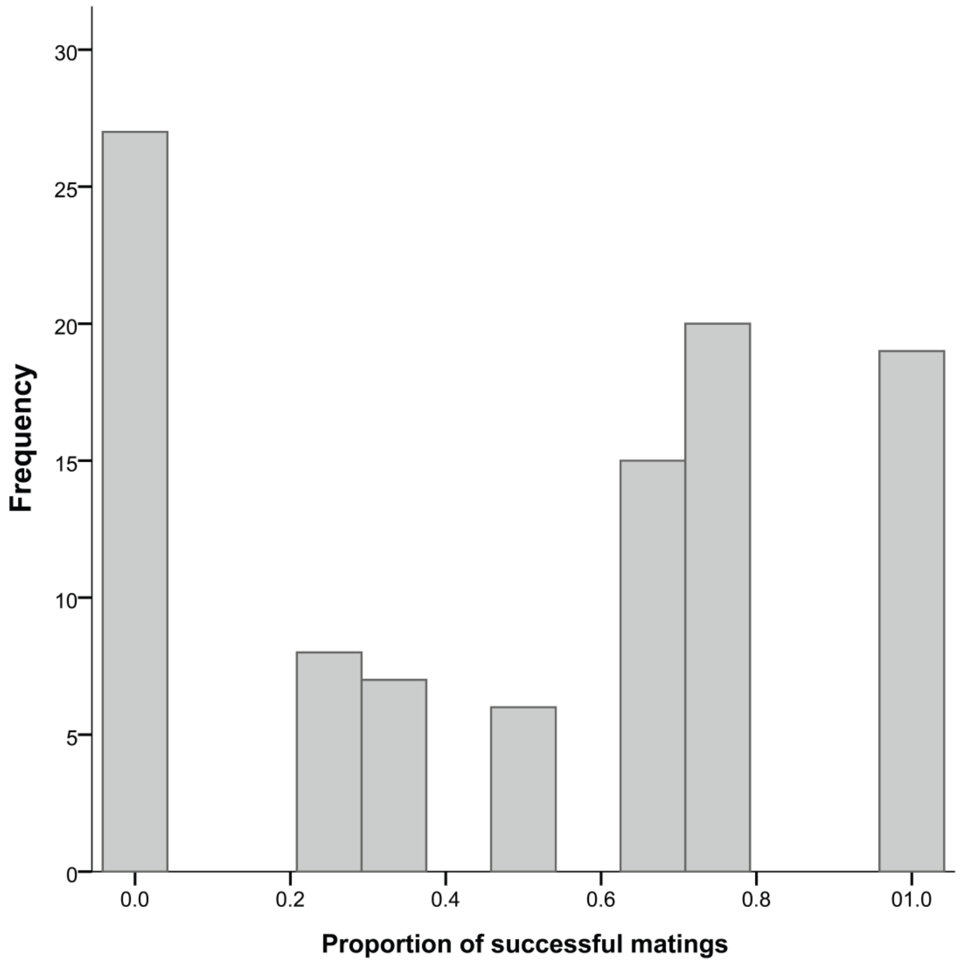

Fig S1 Frequency distribution of total individual mating success. N= 102, Mean= 0.51

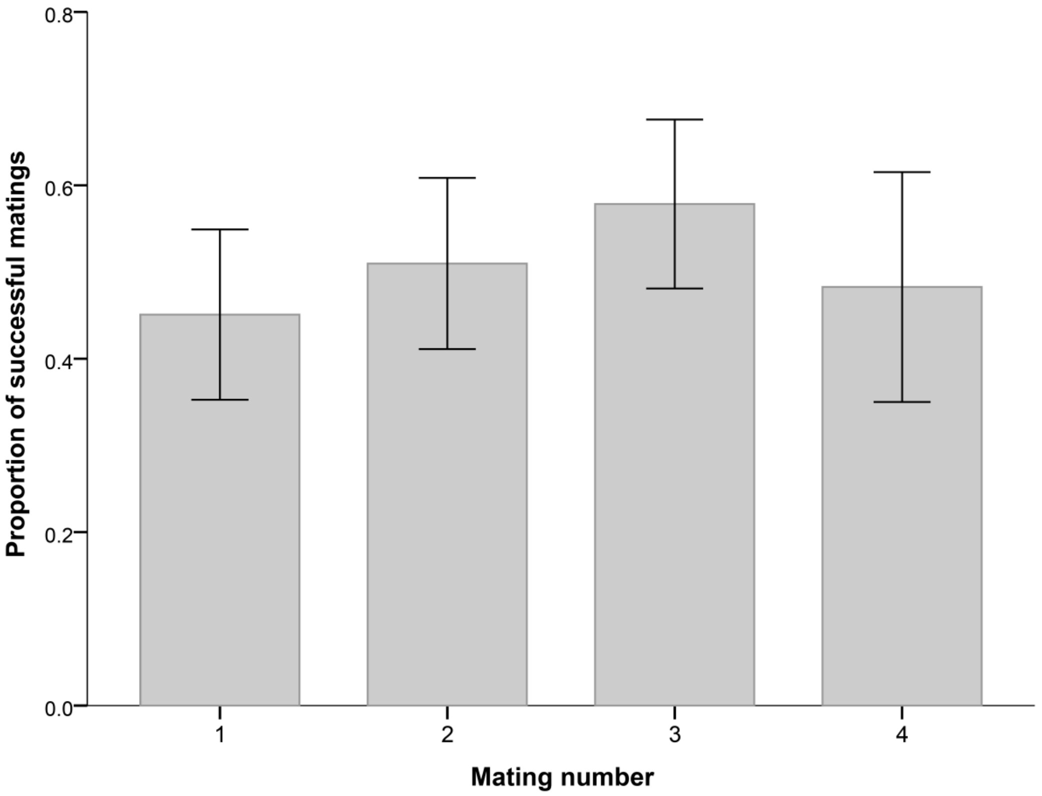

Fig S2 No effect of mating order on mating failure- GLMM, Z= 1.056, P = 0.291. Bars represent 95% CI

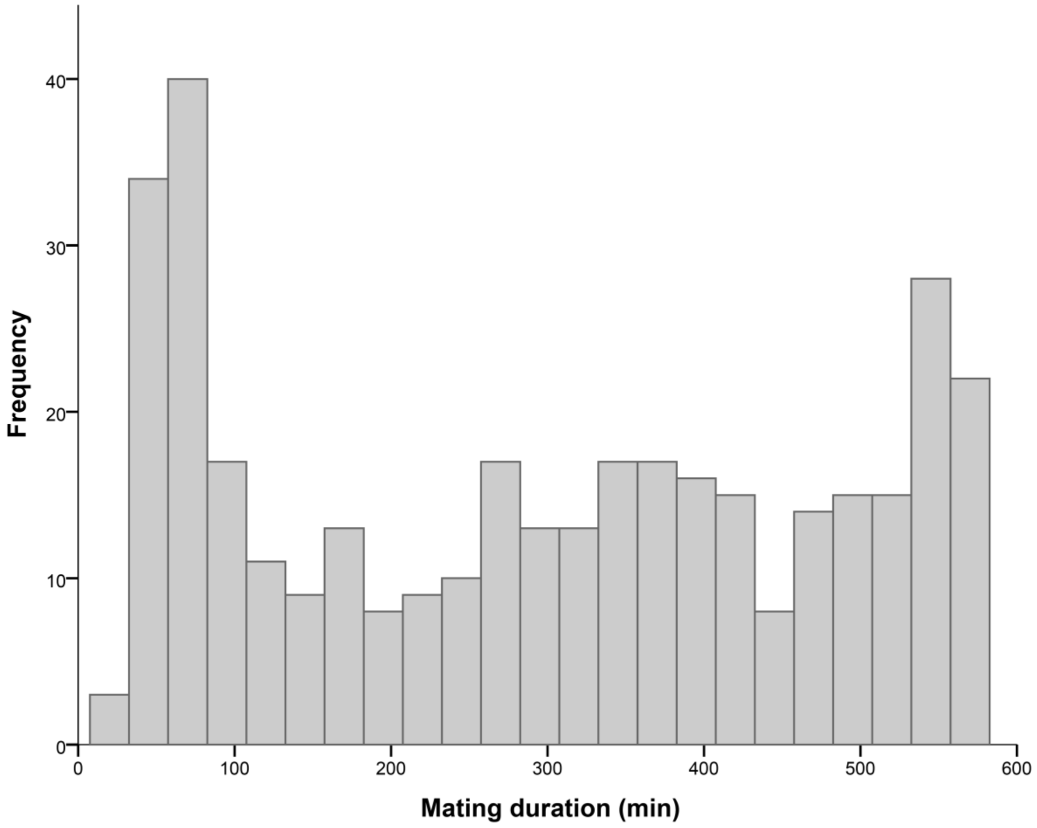

Fig S3 Frequency distribution of mating durations. *N*= 364, mean duration = 295.1 mins. Matings ≤ 295 min classed as 'short', matings > 295min classed as 'long'

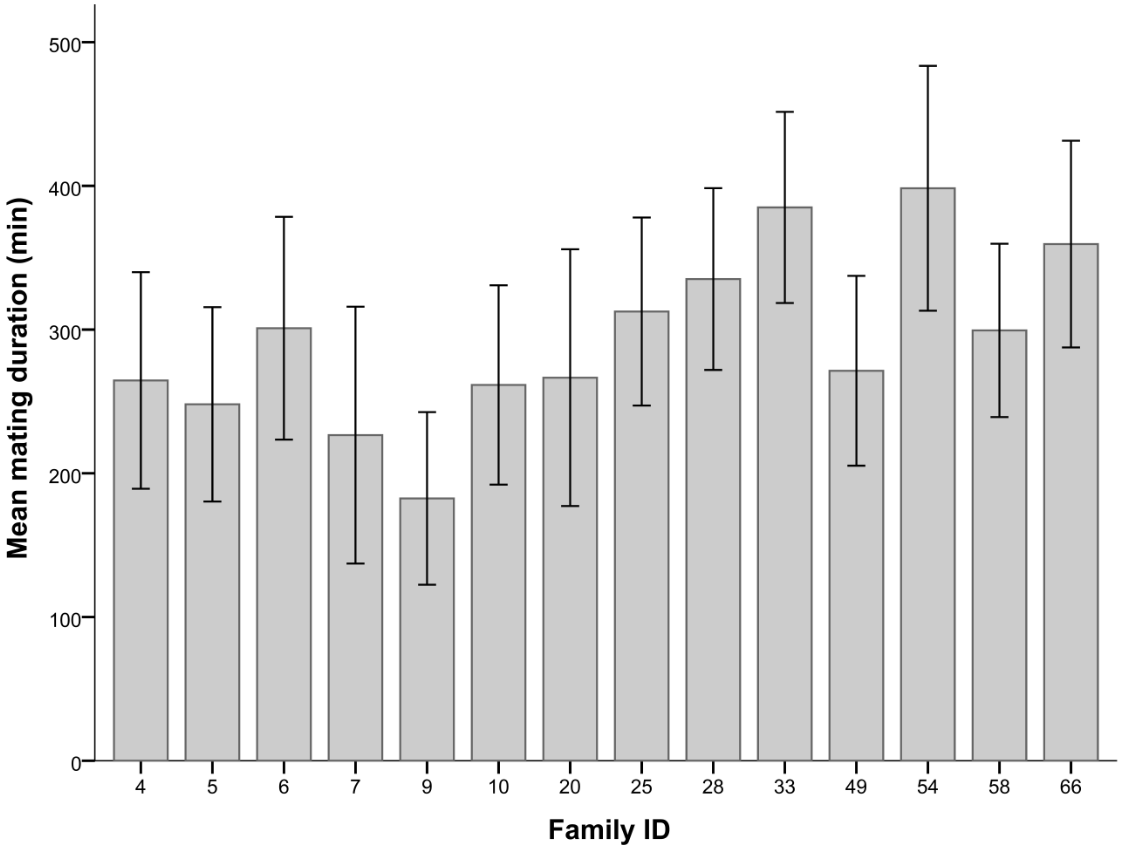

Fig S4 Mean mating duration by family (n=14). Bars represent 95% CI

Reference:

Nakagawa, S., & Schielzeth, H. (2010). Repeatability for Gaussian and non-Gaussian data: a practical guide for biologists. *Biological Reviews of the Cambridge Philosophical Society*, *85*(4), 935–56.
